# Comparison of Methods to Assess Adolescent Gender Identity in the ABCD Study

**DOI:** 10.1001/jamapediatrics.2023.4678

**Published:** 2023-11-06

**Authors:** Sarahjane L. Dube, Michelle M. Johns, Leah Robin, Elizabeth Hoffman, Alexandra S. Potter

**Affiliations:** 1 Department of Psychiatry, Larner College of Medicine, University of Vermont, Burlington, Vermont; 2 Center on Equity Research, NORC at the University of Chicago, Atlanta, Georgia; 3 Division of Adolescent and School Health, Centers for Disease Control and Prevention, Atlanta, Georgia; 4 National Institute on Drug Abuse, Bethesda, Maryland

## Abstract

This cross-sectional study assesses the reliability and validity of methods used to capture gender identity in Adolescent Brain Cognitive Development (ABCD) Study participants.

While reliable and valid methods to assess gender identity exist for adults, their utility and feasibility are less well understood for adolescents. For adults, the 2-step method, asking sex assigned at birth and current gender identity, is recommended for clinical settings and population-based studies^1^ as it accurately captures gender diverse (GD) identities that differ from those typically associated with assigned sex (eg, transgender, nonbinary).^2,3,4^

## Methods

This cross-sectional study was approved by the University of California, San Diego central institutional review board, and parent and child written informed consent or assent was obtained. The study followed the STROBE reporting guideline.

The Adolescent Brain Cognitive Development Study is among the first nationally to use the 2-step method with adolescents. At the 3-year follow-up visit (age, 12-13 years), youths were asked to report their assigned sex on their birth certificate (male, female, don’t know [DK], refuse to answer) and current gender identity (boy, girl, another gender [eg, nonbinary], I don’t understand the question [IDU], refuse to answer). A separate measure asked, “Are you transgender? Yes, maybe, no, IDU, refuse to answer.”^5^ Data from the 5.0 release, in the National Institute of Mental Health Data Archive, include the full cohort at the 3-year visit and approximately half at the 4-year visit. Responses to both 2-step items (10 273 youths at 3 years and 4720 at 4 years) were analyzed for feasibility (rates of refusal, DK, IDU) and to compare methods of identifying GD adolescents. Data analysis was performed from June 21 to July 14, 2023, using SPSS, version 29.0 (IBM Corporation). A 2-sided *P* < .05 was considered significant by χ^2^ test.

## Results

Rates of refusal and DK or IDU were 1% or less and less than 2%, respectively, for each item (Table 1), suggesting broad acceptability. The transgender item had rates of refusal of less than 1% and IDU less than 2.5% at both time points (Table 2). These rates are consistent with other items across the protocol. Youth-reported assigned sex matched parent report (collected at enrollment) for 99.9% of the sample (12 at 3 years and <10 at 4 years did not match), suggesting adolescent comprehension.

**Table 1.** Cross Tabulation of 2-Step Method (Assigned Sex by Gender Identity) at 3- and 4-Year Visits

	Youths, No. (%)							
	3-y Visit (n = 10 273; aged 12-13 y)				4-y Visit (n = 4720; aged 13-14 y)			
	Male (n = 5251)	Female (n = 4793)	DK (n = 199)	Refuse (n = 30)	Male (n = 2440)	Female (n = 2182)	DK (n = 42)	Refuse (n = 11)
Boy	5173 (99)	60 (1)	110 (55)	10 (33)	2410 (99)	39 (2)	23 (55)	<10^b^
Girl	22 (0)	4491 (94)	73 (37)	<10^b^	<10^b^	1977 (89)	13 (31)	<10^b^
Another^a^	26 (1)	179 (4)	0	0	18 (1)	169 (8)	<10^b^	<10^b^
IDU	13 (0)	23 (1)	<20^c^	<10^b^	<10^b^	<10^b^	<10^b^	<10^b^
Refuse	17 (0)	40 (1)	<10^b^	16 (53)	<10^b^	<40^c^	<10^b^	<10^b^

Abbreviations: DK, don’t know; IDU, “I don’t understand the question.”

^a^ The full response option is “another gender (eg, nonbinary).”

^b^ Frequencies less than 10 are not specified to protect privacy and confidentiality.

^c^ Secondary suppression to ensure that the less than 10 cell cannot be recalculated through subtraction.

**Table 2.** Cross Tabulation of 2-Step Gender Identity by Transgender Item at 3- and 4-Year Visits

	Youths, No. (%)							
	3-y Visit (n = 10 273; aged 12-13 y)				4-y Visit (n = 4270; aged 13-14 y)			
	GD boy or girl (n = 82)	GD another gender (eg, nonbinary) (n = 205)	Not GD boy or girl (n = 9664)^a^	DK, IDU, or refuse (n = 322)	GD boy or girl (n = 47)	GD another gender (eg, nonbinary) (n = 187)	Not GD boy or girl (n = 4387)^a^	DK, IDU, or refuse (n = 95)
Yes	55 (67)	42 (21)	<10^b^	<10^b^	41 (87)	45 (24)	<10^b^	<10^b^
Maybe	<10^b^	46 (22)	42 (0)	<20^c^	<10^b^	44 (24)	21 (1)	<10^b^
No	19 (23)	111 (54)	9396 (97)	215 (67)	<10^b^	94 (49)	4321 (99)	55 (58)
IDU	<10^b^	<10^b^	201 (2)	48 (15)	0	<10^b^	39 (1)	<10^b^
Refuse	<10^b^	<10^b^	<30^c^	38 (12)	0	<10^b^	<10^b^	22 (23)

Abbreviations: DK, don’t know; GD, gender diverse; IDU, “I don’t understand the question.”

^a^ Not GD are youths whose gender identity is that typically associated with their assigned sex (cisgender).

^b^ Frequencies less than 10 are not specified to protect privacy and confidentiality.

^c^ Secondary suppression to ensure that the less than 10 cell cannot be recalculated through subtraction.

The 2-step method characterized 287 youths (2.8%) at 3 years and 238 (5.0%) at 4 years as GD (Table 1). Across time points, more GD youths were assigned female than male (447 vs 74; *P* < .001), and 392 of the 521 (75%) GD youths selected the nonbinary option. For 4643 youths with data at both time points, 55 of 75 (73%) GD youths at 3 years were also GD at 4 years.

The transgender item characterized 216 (2.1%) and 168 (3.6%) youths as transgender (yes, maybe) at each visit (Table 2). Importantly, the 2-step method categorized 1.3 to 1.4 times more GD participants. Approximately one-half of youths who selected the nonbinary option responded no to the transgender item vs less than one-quarter boy or girl GD youths, suggesting that the 2-step method may be more sensitive and inclusive for GD adolescents than only asking about transgender status.

Of the participants not identified as GD using the 2-step method, 68 (0.5%) endorsed the transgender item (primarily “maybe”) (Table 2). Though a small proportion, these youths may be unsure of their current gender identity; may identify explicitly as transgender, transgender boy, or transgender girl and, without that option, chose the gender response congruent with their sex; or responded in error.

## Discussion

The findings point to the feasibility of using the 2-step method in adolescents as young as 12 years. Consistent with previous reports,^6^ youths seemed to understand the assigned sex question, and no negative outcomes associated with data collection were detected. Providing a nonbinary option may be important for inclusivity and to capture GD youths who do not identify as transgender (43% of GD youth in this sample). This work lacked, but would benefit from, cognitive interviews and direct comparisons of the gender identity question with one that includes transgender, unsure, and check-all-that-apply response options.

## Notes

Supplement.
Data Sharing Statement
