# Supplementary material for: Comparison of Methods to Assess Adolescent Gender Identity in the ABCD Study
Source: JAMA Pediatr. 2023 Nov 6;178(1):86–8. doi: 10.1001/jamapediatrics.2023.4678 (PMC10628840; doi:10.1001/jamapediatrics.2023.4678)
Supplement: Supplement. — Data Sharing Statement [file jamapediatr-e234678-s001.pdf]

Data Sharing Statement

Dube. Comparison of Methods to Assess Adolescent Gender Identity in the ABCD Study. *JAMA Pediatr*. Published November 06, 2023. doi:10.1001/jamapediatrics.2023.4678

Data
Data available: Yes
Data types: Deidentified participant data, Data dictionary
How to access data: https://nda.nih.gov/abcd/
When available: beginning date: 06-20-2023

Supporting Documents
Document types: None

Additional Information
Who can access the data: Qualified researchers with a completed Data Use Certification may access ABCD shared data on the NIMH Data Archive.
Types of analyses: Any research-related need.
Mechanisms of data availability: The data repository houses all data generated by the Adolescent Brain Cognitive Development (ABCD) Study. Data Access Requests to ABCD will be reviewed by the ABCD Data Access Committee. Approved users will have access to ABCD data repositories. The ABCD data repository grows and changes over time. The ABCD data used in this report came from NIMH Data Archive Digital Object Identifier 10.15154/8873-zj65. DOIs can be found at https://nda.nih.gov/study.html?id=2147.
Any additional restrictions: You must have a research-related need to access the data. You must be associated with an NIH-recognized research institution, defined as an institution registered in the NIH electronic research administration system (eRA Commons) and have the approval of an authorized signatory official of that institution. Your institution must have an active Federalwide Assurance (FWA).
